# Precision Medicine for Blood Glutamate Grabbing in Ischemic Stroke

**DOI:** 10.3390/ijms25126554

**Published:** 2024-06-14

**Authors:** Pablo Hervella, Ana Sampedro-Viana, Sabela Fernández-Rodicio, Manuel Rodríguez-Yáñez, Iria López-Dequidt, José M. Pumar, Antonio J. Mosqueira, Marcos Bazarra-Barreiros, María Teresa Abengoza-Bello, Sara Ortega-Espina, Alberto Ouro, María Pérez-Mato, Francisco Campos, Tomás Sobrino, José Castillo, Maria Luz Alonso-Alonso, Ramón Iglesias-Rey

**Affiliations:** 1 Neuroimaging and Biotechnology Laboratory (NOBEL), Clinical Neurosciences Research Laboratory (LINC), Health Research Institute of Santiago de Compostela (IDIS), 15706 Santiago de Compostela, Spain; pablo.hervella.lorenzo@sergas.es (P.H.); ana.sampedro@rai.usc.es (A.S.-V.); sabela.fernandez.rodicio@rai.usc.es (S.F.-R.); josemanuel.pumar@usc.es (J.M.P.); drmosqueiramartinez@gmail.com (A.J.M.); marcos.bazarra.barreiros@sergas.es (M.B.-B.); maria.teresa.abengoza.bello@sergas.es (M.T.A.-B.); sara.ortega@rai.usc.es (S.O.-E.); jose.castillo.sanchez@sergas.es (J.C.); 2 Stroke Unit, Department of Neurology, Hospital Clínico Universitario, 15706 Santiago de Compostela, Spain; manuel.rodriguez.yanez@sergas.es; 3 Department of Neurology, Hospital Clínico Universitario de Ferrol, 15405 Ferrol, Spain; iriaalejandralopez@googlemail.com; 4 Department of Neuroradiology, Hospital Clínico Universitario, Health Research Institute of Santiago de Compostela (IDIS), 15706 Santiago de Compostela, Spain; 5 NeuroAging Group (NEURAL), Clinical Neurosciences Research Laboratory (LINC), Health Research Institute of Santiago de Compostela (IDIS), 15706 Santiago de Compostela, Spain; alberto.ouro.villasante@sergas.es (A.O.); tomas.sobrino.moreiras@sergas.es (T.S.); 6 Centro de Investigación Biomédica en Red de Enfermedades Neurodegenerativas, Instituto de Salud Carlos III, 28029 Madrid, Spain; 7 Translational Stroke Laboratory (TREAT), Clinical Neurosciences Research Laboratory (LINC), Health Research Institute of Santiago de Compostela (IDIS), 15706 Santiago de Compostela, Spain; maria.perez.mato@sergas.es (M.P.-M.); francisco.campos.perez@sergas.es (F.C.)

**Keywords:** biomarkers, blood–brain barrier, glutamate grabbers, glutamate oxaloacetate transaminase, ischemic stroke, sTWEAK

## Abstract

Glutamate grabbers, such as glutamate oxaloacetate transaminase (GOT), have been proposed to prevent excitotoxicity secondary to high glutamate levels in stroke patients. However, the efficacy of blood glutamate grabbing by GOT could be dependent on the extent and severity of the disruption of the blood–brain barrier (BBB). Our purpose was to analyze the relationship between GOT and glutamate concentration with the patient’s functional status differentially according to BBB serum markers (soluble tumor necrosis factor-like weak inducer of apoptosis (sTWEAK) and leukoaraiosis based on neuroimaging). This retrospective observational study includes 906 ischemic stroke patients. We studied the presence of leukoaraiosis and the serum levels of glutamate, GOT, and sTWEAK in blood samples. Functional outcome was assessed using the modified Rankin Scale (mRS) at 3 months. A significant negative correlation between GOT and glutamate levels at admission was shown in those patients with sTWEAK levels > 2900 pg/mL (Pearson’s correlation coefficient: −0.249; *p* < 0.0001). This correlation was also observed in patients with and without leukoaraiosis (Pearson’s correlation coefficients: −0.299; *p* < 0.001 vs. −0.116; *p* = 0.024). The logistic regression model confirmed the association of higher levels of GOT with lower odds of poor outcome at 3 months when sTWEAK levels were >2900 pg/mL (OR: 0.41; CI 95%: 0.28–0.68; *p* < 0.0001) or with leukoaraiosis (OR: 0.75; CI 95%: 0.69–0.82; *p* < 0.0001). GOT levels are associated with glutamate levels and functional outcomes at 3 months, but only in those patients with leukoaraiosis and elevated sTWEAK levels. Consequently, therapies targeting glutamate grabbing might be more effective in patients with BBB dysfunction.

## 1. Introduction

The aging of the population in developed countries, along with relevant advances in medical care, has increased the number of people who overcome serious illnesses, such as stroke. However, this involves an increase in morbidity [1]. Although stroke remains the second-leading cause of death worldwide and the third-leading cause of death and disability combined [2,3], the increase in survival rate with severe disabilities represents a remarkable cost, not only in terms of health burden but also in the quality of life of patients and their caregivers [4,5]. It should be highlighted that the development of stroke units in which patients receive specialized stroke care has played a significant role in this improvement of outcomes [6]. The current therapeutic options for acute ischemic stroke, the main contributor to the global incidence of stroke [2], are mechanical thrombectomy, thrombolytic therapy, or a combination of both [7]. However, these therapeutical options still present notable limitations, including the number of eligible patients and the time lapse from stroke onset to specialized assistance [8]. Additionally, recanalization success is not always correlated with clinical improvement [9,10,11,12,13]. Neuroprotection was proposed as a potential option to improve the poor outcomes of patients. Several targets have been studied; unfortunately, despite the promising results obtained in preclinical assays, numerous neuroprotective drugs have failed to be transferred to clinical practice [14,15].

Several factors have been described to be involved in this failure, such as the differences in the time window selected in preclinical and clinical studies and the heterogeneity of patients included in clinical trials, among others [16]. All this evidence reinforces the increasing interest in precision medicine in stroke, which integrates blood biomarkers and clinical and neuroimaging data [17,18].

Among others, it was demonstrated that high serum glutamate concentration could be considered a blood biomarker of poor outcomes in stroke patients [19,20,21,22,23]. Glutamate plays an important role in neural injury during ischemia-induced excitotoxicity [24]. Recently, glutamate grabbers or scavengers have been proposed to prevent a cascade of events triggered by excessive extracellular glutamate [25,26,27].

In physiological conditions, the excess of extracellular glutamate is transported into endothelial cells, belonging to the blood–brain barrier (BBB), from where it diffuses to blood due to the difference in concentration [28]. However, in ischemic conditions, structural integrity of the BBB is affected, promoting an increase in the permeability [29]. Based on these data, we hypothesize that glutamate grabbing through a normal endothelium would not be enough to decrease excitotoxicity in the penumbra zones. Therefore, the efficacy of blood glutamate grabbing could depend on the extent and severity of the disruption of the BBB. Soluble tumor necrosis factor-like weak inducer of apoptosis (sTWEAK) plays a role in triggering neuroinflammation and BBB breakdown during ischemic stroke [30]. This makes it a potential serum marker of BBB disruption. Additionally, leukoaraiosis, defined as an abnormality in the white matter substance detected on computed tomography (CT) or magnetic resonance image (MRI) [31], has been previously associated with sTWEAK levels by our group [32].

On the other hand, it is well known that glutamate oxaloacetate transaminase (GOT) is a glutamate grabber. This enzyme catalyzes the reversible transformation of glutamate and oxaloacetate to α–ketoglutarate and aspartate [25,33]. It has been shown that high blood levels of GOT are associated with better outcomes both in animal models [34,35] and ischemic stroke patients [36,37].

The main purpose of this study was to analyze the relationship between GOT and glutamate concentration, determined in plasma at admission, and the patient’s functional status at 3 months. This analysis was performed differentially in patients with serum (sTWEAK) and neuroimaging (leukoaraiosis) markers of BBB disruption determined at admission.

## 2. Results

### 2.1. Sample Description

After applying the inclusion and exclusion criteria established, 906 patients were finally enrolled for this analysis. The flowchart for patient screening from Biobanco Ictus del Complejo Hospitalario Universitario de Santiago (BICHUS) is shown in Figure S1. The sex distribution of the sample was 51% males vs. 49% females, and the mean age was 72.2 ± 12.1 years. These patients were classified according to the TOAST (Trial of Org 10,172 in Acute Stroke Treatment) criteria as atherothrombotic (20.5%), cardioembolic (37%), lacunar (9.4%), and indeterminate (31.1%). 5.4% of patients have undergone thrombectomy and 14.5% have intravenous fibrinolysis.

### 2.2. Demographic and Clinical Features Associated with Poor Functional Outcome at 3 Months

The bivariate analysis of demographic and clinical variables collected from the ischemic stroke patients showed that the time lapse between the onset of symptoms and the emergency assistance is an essential variable (277.8 ± 214.5 min vs. 269.9 ± 180.7 min; *p* = 0.002) for clinical progress. Similarly, the presence of leukoaraiosis in the neuroimaging studies was associated with poor outcomes (*p* < 0.0001). Concerning potential biomarkers measured in blood samples, results showed that the levels of GOT at admission were significantly lower in those patients with poor functional outcomes at 3 months (39.7 ± 13.3 U/L vs. 22.6 ± 10.3 U/L; *p* < 0.0001). Contrary to this, the levels of glutamate (134.2 ± 112.5 μM/mL vs. 227.1 ± 123.8 μM/mL; *p* = 0.036) and sTWEAK (3143.2 ± 2677.1 pg/mL vs. 5828.6 ± 3248.9 pg/mL; *p* = 0.002) were significantly higher in these patients. There were also significant differences between those with good and poor outcomes in terms of age (*p* = 0.003), alcohol consumption (*p* = 0.004), sex, smoking, and atrial fibrillation (all *p* < 0.0001). Additionally, those patients who showed higher temperature (*p* = 0.029), blood glucose, and leukocyte count at admission (both *p* < 0.0001) showed worse outcomes at 3 months. TOAST criteria and higher National Institute of Health Stroke Scale (NIHSS) scores at admission (both *p* < 0.0001) were also significantly associated with poorer long-term outcomes (Table 1).

### 2.3. GOT and sTWEAK Association with Poor Functional Outcome at 3 Months

**Figure 1:**
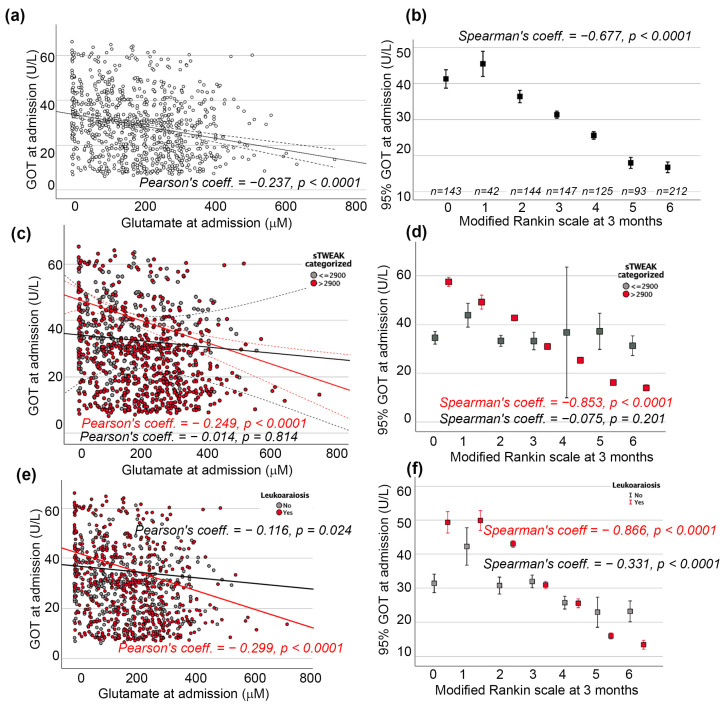
Correlation coefficient analysis between glutamic-oxaloacetic transaminase (GOT) and glutamate levels at admission (**a**) without patient categorization, (**c**) by soluble tumor necrosis factor-like weak inducer of apoptosis (sTWEAK) level, and (**e**) by leukoaraiosis. Correlation coefficient analysis between GOT and modified Rankin scale at 3 months (**b**) without patient categorization, (**d**) by soluble tumor necrosis factor-like weak inducer of apoptosis (sTWEAK) level, and (**f**) by leukoaraiosis.

A significant negative correlation between GOT and glutamate levels was found at admission (Pearson’s correlation coefficient: −0.237; *p* < 0.0001; Figure 1a). It was also observed that lower levels of GOT were correlated with higher scores on the modified Rankin Scale (mRS) and, therefore, with higher rates of morbidity and mortality at 3 months (Figure 1b).

Regarding the other blood biomarkers, sTWEAK, according to ROC curve analysis, can discriminate between patients with poor outcomes and those with good outcomes at 3 months with a sensitivity of 88% and a specificity of 70% (Figure S2a). The cut-off for it was established at 2900 pg/mL (Figure S2a). Results showed a higher percentage of patients with sTWEAK levels higher than 2900 pg/mL for all the ischemic stroke types classified following the TOAST criteria, except for lacunar strokes (Figure S2b).

When the correlation between GOT and glutamate levels at admission was reanalyzed according to the sTWEAK serum level, the significant negative correlation between them was confirmed in those patients with sTWEAK > 2900 pg/mL (Pearson’s correlation coefficient: −0.249; *p* < 0.0001) but not for patients with lower levels (Figure 1c). Similarly, the significant negative correlation between GOT and mRS was retained only in patients with high levels of sTWEAK (Figure 1d). The logistic regression model confirmed this independent association of higher levels of GOT with lower odds of poor functional outcome at 3 months when sTWEAK was >2900 pg/mL (OR: 0.41; CI 95%: 0.28–0.68; *p* < 0.0001; Table 2).

### 2.4. GOT and Leukoaraiosis Association with Poor Functional Outcome at 3 Months

As shown in Figure S3, GOT and sTWEAK showed a significant association (*p* < 0.001) with the degree of leukoaraiosis observed in the neuroimaging studies. Meanwhile, the relationship with sTWEAK was positive (Figure S3a), and a negative correlation with GOT was found (Figure S3b).

Additionally, the correlation between GOT and glutamate levels at admission was re-analyzed according to the leukoaraiosis degree; the significant negative correlation between them was confirmed in both groups with (Pearson’s correlation coefficient: −0.299; *p* < 0.0001) and without leukoaraiosis (Pearson’s correlation coefficient: −0.116; *p* = 0.024) (Figure 1e). Consequently, the significant negative correlation between GOT and mRS was maintained regardless of the presence or absence of leukoaraiosis (Figure 1f). However, the logistic regression model only confirmed this independent association of higher levels of GOT with lower odds of poor functional outcome at 3 months in the presence of this neuroimaging biomarker (OR: 0.75; CI 95%: 0.69–0.82; *p* < 0.0001; Table 3).

## 3. Discussion

In this study, the association between the enzyme GOT with glutamate at admission and with the patient’s functional status at 3 months was evaluated by classifying patients according to two biomarkers of BBB disruption, sTWEAK and leukoaraiosis. The inverse correlations between GOT with glutamate and with functional status previously described were supported by present results [36,37].

Precision medicine is defined by the National Institutes of Health as “an innovative approach that takes into account individual differences in patients’ genes, environments, and lifestyles” [38]. The interest in this field has increasingly grown in the last decades for a wide range of diseases [39], including cerebrovascular diseases [18]. The tools used for this purpose are very varied [40]. Different blood biomarkers have been reported as useful tools for diagnosis, treatment decisions, and outcome prediction in stroke [41]. This type of biomarker has the advantage that it can be incorporated into daily clinical practice more easily than others since blood collection is a routine activity. Blood is the most common sample evaluated in recent biomarker trials for stroke [42].

On the other hand, currently there is only one clinical trial based on glutamate grabbers, riboflavin (vitamin B2) (NCT02446977; completed), which was developed by our group. Although the results of this clinical trial showed a demonstration of the efficacy of this blood glutamate grabber in the treatment of patients with stroke [27], a precision medicine approach was not followed, which is a point to be improved in the future. Taking in mind the relevance of maintaining BBB integrity for brain health and cerebrovascular diseases, we hypothesized that biomarkers of its disruption would improve not only the clinical approach of current treatments but also will be an advantageous tool considered for the selection of patients in future clinical trials. In the present study, we selected a blood biomarker, i.e., sTWEAK, and a neuroimaging marker, leukoaraiosis. Both would be easily transferred into clinical practice since blood sample analysis and neuroimaging studies are usual in stroke patients approaches.

The sTWEAK, a cytokine involved in different cellular activities such as proliferation, migration, differentiation, apoptosis, angiogenesis, and inflammation [43], has been identified as a potential biomarker of BBB disruption based on its implication in disruption of the structure of BBB and the increment of its permeability. Thus, in 2005 Polavarapu et al. [44] showed that intracerebral injection of TWEAK in wild-type mice led to disruption in the structure of the neurovascular unit and to an increase of BBB permeability. In this study, the inhibition of its activity resulted in a significant preservation of the integrity of the neurovascular unit and a diminution of the BBB permeability. The results of Zhang et al. in 2007 [45] by the inhibition of the TWEAK-fibroblast growth factor-inducible 14 (Fn14) axis in an animal model of middle cerebral artery occlusion also support the role of TWEAK in the disruption of the BBB. Finally, in 2013, Stephan et al. [46] showed that TWEAK increased permeability in an in vitro model of BBB with endothelial cells.

High levels in sTWEAK serum have been associated with poor outcomes not only in cerebrovascular diseases, such as ischemic stroke [13,47], intracerebral hemorrhage [48,49], but also in traumatic brain injury [50]. Similarly, we found poor outcomes associated with higher serum levels of sTWEAK. Our results have allowed us to establish a cut-off value of 2900 pg/mL at admission to differentiate good and bad outcomes at 3 months with a sensitivity of 88% and a specificity of 70%. Although the cut-off value is an essential fact for biomarker research, it has been reported that different cut-off values of sTWEAK according to the main variable studied. Thus, Hervella et al. [13] reported a cut-off value of 7000 pg/mL for stroke recurrence, and da Silva-Candal et al. [47,49] determined a cut-off value of 5600 pg/mL for hematoma growth and 6700 pg/mL for poor outcome in reperfused ischemic stroke patients, whereas Comertpay et al. [51] stated a cut-off value of barely 995.5 pg/mL for the diagnosis of acute ischemic stroke. Our cut-off value of 2900 pg/mL was taken into consideration when analyzing the relationship of GOT and glutamate; this was only significant in those patients whose serum levels of sTWEAK were higher than the cut-off established. Therefore, sTWEAK could play a key role in the association of high levels of GOT described in stroke patients with good outcomes [36,37].

Similar results were obtained when the presence of leukoaraiosis was taken into consideration. Leukoaraiosis is defined as an abnormality in the white matter substance detected on CT or MRI [31]. The implication of the dysfunction of BBB in the pathogenies of white matter lesions is suggested by several studies [52,53,54]. Additionally, a previous study of our group showed an association between sTWEAK levels and leukoaraiosis degree [32]. Based on these facts, leukoaraiosis could also be considered a biomarker of BBB dysfunction. Although leukoaraiosis did not initially appear to affect the negative correlation between GOT and glutamate, adjusted regression analysis did show that leukoaraiosis, like sTWEAK, could play a role in this enzyme-amino acid relationship. This supports the leukoaraiosis useless as a differential criterion between poor and good outcomes previously described in stroke patients [13,47,55,56].

Therefore, our results showed that BBB disruption could be a key factor in the effectiveness of glutamate grabber therapy. The breakdown of BBB during stroke is due to the triggering of cascades that lead to an increase in permeability and loss of integrity as a result of the changes in endothelial cells [29,57]. It is well established that endothelial cells are involved in the elimination of glutamate from the brain to blood by facilitating the BBB transport [58], where glutamate is metabolized by a glutamate grabber. The changes suffered by endothelial cells and the subsequent BBB disruption could promote the brain-to-blood glutamate efflux, promoting the effectivity of glutamate grabbers. To determine the specific mechanisms involved in this process, further in vitro and in vivo studies would be necessary.

This study has been focused on GOT as a glutamate grabber. However, it should be noted that there are other strategies for lowering blood glutamate, including another enzyme, glutamate-pyruvate transaminase (GPT), and hemodialysis and peritoneal dialysis [25]. Similarly to GOT, high levels of GPT have been associated with good outcomes both in animal models [59] and ischemic stroke patients in whom this association was weaker than for GOT [37]. Regarding hemodialysis and peritoneal dialysis, studies in patients with renal diseases have shown that both methods effectively reduced levels of glutamate in the blood, which makes them a potential tool for preventing excitotoxicity in stroke [60,61]. There are two clinical trials (NCT04297345 and NCT03454867) testing this hypothesis of blood filtration for clearance of glutamate. Finally, peritoneal dialysis. Therefore, future studies about the association of these glutamate-lowering strategies with BBB disruption biomarkers could also be relevant for advancing in precision medicine in ischemic stroke.

Finally, our study has several limitations. First, this is a single-center study. These results should be validated by patients from other databases from different centers. Secondly, those related to its retrospective design. Thirdly, the serum levels of the biomarkers were only measured at admission, except for sTWEAK, which was also analyzed at 3 months. On the other hand, the strengths of this work include the unbiased selection of patients, the large sample size included, and the blind analysis.

## 4. Materials and Methods

### 4.1. Standard Protocol Approval and Patient Consent

This research was conducted following the principles of the Declaration of Helsinki of the World Medical Association and approved by the Research Ethics Committee of Santiago (project identification code 2019/616). Informed consent was obtained from each patient or their relatives after fully explaining the procedures.

### 4.2. Study Design

This is a retrospective observational study of patients with stroke admitted to the Stroke Unit of the Hospital Clínico Universitario of Santiago de Compostela (Spain) between January 2008 and December 2018. They were consecutively and prospectively registered in an approved data bank, BICHUS. All patients admitted to the Stroke Unit were treated by expert neurologists according to Spanish Neurological Society protocol.

Upon admission to the Stroke Unit, the intensity of the neurological deficit was determined by NIHSS [62]. Regarding functional outcome, it was assessed using mRS [63] at 3 months ± 15 days (face-to-face in 80.8% of the sample). A mRS > 2 was classified as a poor outcome. Internationally certified neurologists evaluated both scales. Stroke etiology was classified according to TOAST criteria [64].

MRI or CT studies were performed at admission. Leukoaraiosis was defined as low density on CT and hyperintensity on T2-weighted or FLAIR MRI. The presence and severity of leukoaraiosis were assessed using the Fazekas scale with a total score from 0 to 6 (grade I, 1–2; grade II, 3–4; and grade III, 5–6) [65]. These neuroimaging studies were performed using the same protocol and supervised by the same neuroradiologists.

### 4.3. Inclusion and Exclusion Criteria

Inclusion criteria for this analysis were as follows: (1) authorization for the anonymous use of individuals’ data for research purposes; (2) MRI or CT study at admission. Exclusion criteria were: (1) patients without a minimum follow-up (face-to-face or telephone) of 3 months; (2) institutionalized patients; (3) comorbidity and life expectancy of less than 1 year; (4) lack of subsequent diagnostic confirmation; (5) absence of blood tests on the admission of the patient with the determination of GOT; and (6) unavailability of frozen plasma sample for analysis of glutamate and sTWEAK. The exclusion of patients did not imply bias in the main clinical variables in relation to the selection of patients valid for this study (Table S1).

Demographic and clinical features of patients analyzed in the present study were obtained from the anamnesis and the medical history.

### 4.4. Biochemical and Hematological Analysis

Biochemistry, hematology, and coagulation assays from blood samples collected during the first 6 h after admission were performed in the hospital’s central laboratory. In addition, we evaluated the sTWEAK in the patients’ serum at admission and after 3 months. Although these analyses were not performed simultaneously, they used the same standardized methods, and the same researchers supervised them.

Venous blood samples were collected in vacutainer tubes (Becton Dickinson, San Jose, CA, USA) in the first 6 h after symptom onset (always after the administration of the thrombolytic bolus). After allowing the samples to coagulate for 60 min, the blood samples were centrifuged at 3000× *g* for 10 min at 4 °C, and the serum was immediately aliquoted, frozen, and stored at −80 °C until analysis.

The determination of sTWEAK serum concentration was conducted without knowledge of the clinical data, using the enzyme-linked immunosorbent assay (ELISA) technique as per the manufacturer’s instructions (ThermoFisher Scientific, Waltham, MA, USA). The minimum assay sensitivity was 40 pg/mL, and both intra- and inter-assay coefficients of variation were kept below 10% and 12%, respectively.

Serum glutamate concentration was analyzed through high-performance liquid chromatography (1260 Infinity II, Agilent Technologies, Santa Clara, CA, USA) employing the AccQ-Tag™ Precolumn derivatization method for amino acid analysis (Waters, Milford, MA, USA), following a previously outlined method [66].

GOT levels were measured using an automated auto-analyzer (ADVIA 2400, Bayer Diagnostics, Leverkusen, Germany).

All the samples were anonymized using a code so that the researcher who developed the analysis had no information about the clinical status through the follow-up period, avoiding biases in the analysis.

### 4.5. Statistical Analysis

Categorical variables were described with frequency and percentage. For this descriptive study, the quantitative variables were described with the mean ± standard deviation, or the median and interquartile range, according to the type of distribution determined by the Kolmogorov–Smirnov test for a sample with the significance correction of Lilliefors.

Analysis of variance (ANOVA) was used to compare differences between more than two groups. According to the nature of the contrast variable and its adjustment to normality, the significance of the differences between the two groups was estimated using the chi-square test or Wilcoxon test. Logistic regression analyses were performed to identify those variables independently associated with poor outcomes at 3 months. The results were expressed as odds ratios (ORs) with 95% confidence intervals (95% CI). The analysis of correlations was performed using Pearson’s coefficient and Spearman’s coefficient. The sensitivity and specificity of the biomarkers selected were represented graphically by receiver operating characteristic (ROC) curves. The statistical significance was set at a *p*-value < 0.05. All statistical analyses were performed with SPSS V 29.0.1.0 (171) (IBM, New York, NY, USA).

## 5. Conclusions

The conducted analyses lead to the conclusion that plasma concentrations of GOT are correlated with both glutamate levels and the functional status of patients at the 3-month mark. However, this correlation is observed only in patients with elevated sTWEAK concentrations or those exhibiting leukoaraiosis, indicating BBB disruption. Conversely, the time elapsed since symptom onset and the examined inflammatory biomarkers do not demonstrate a significant relationship with these variables. Consequently, it is plausible to propose that therapies targeting the entrapment of glutamate and reduction of its plasma levels may be more effective in patients experiencing increased BBB permeability and dysfunction. This aspect should be considered for the selection of patients in future clinical trials.

## Figures and Tables

**Table 1:** Bivariate analysis of demographic and clinical features according to functional outcome at 3 months.

	Good Outcome *n* = 329	Poor Outcome *n* = 577	*p*
Latency time	277.8 ± 214.5	269.9 ± 180.7	0.002
Age, years	68.2 ± 13.2	76.1 ± 12.1	0.003
Female gender, %	38.9	54.2	<0.0001
Wake-up stroke, %	12.5	14.6	0.423
Pre-morbid mRS [IQR]	0 [0, 0]	0 [0, 1]	0.055
Arterial hypertension, %	60.8	63.8	0.392
Diabetes mellitus, %	30.4	24.4	0.060
Smoking, %	20.4	12.0	<0.0001
Alcohol consumption, %	9.7	16.6	0.004
Hyperlipidemia, %	30.4	32.9	0.459
Ischemic heart disease, %	10.0	13.3	0.169
Atrial fibrillation, %	12.5	31.9	<0.0001
Heart failure, %	2.7	5.4	0.066
Carotid disease, %	0.9	0.9	0.604
Temperature at admission, °C	37.1 ± 0.7	37.3 ± 0.8	0.029
Glucose at admission, mg/dL	130.7 ± 54.0	154.6 ± 77.7	<0.0001
Leukocytes at admission, ×10^3^/mL	8.9 ± 2.8	10.3 ± 3.7	<0.0001
Fibrinogen at admission, mg/dL	443.9 ± 103.0	478.2 ± 104.8	0.459
LDL cholesterol, mg/dL	119.9 ± 53.2	113.3 ± 47.6	0.203
Sedimentation rate, mm/1st hr	25.5 ± 23.8	33.0 ± 25.1	0.310
GOT at admission, U/L	39.7 ± 13.3	22.6 ± 10.3	<0.0001
Intravenous fibrinolysis, %	14.9	14.2	0.769
Thrombectomy, %	5.8	5.2	0.761
Leukoaraiosis			<0.0001
No, %	50.2	36.2	
Grade I, %	31.0	26.5	
Grade II, %	14.9	18.2	
Grade III, %	4.0	19.1	
TOAST			<0.0001
Atherothrombotic, %	20.1	20.8	
Cardioembolic, %	15.5	49.4	
Lacunar, %	23.4	1.4	
Indeterminate, %	38.6	26.9	
Other, %	2.4	1.6	
NIHSS at admission	5 [3, 11]	17 [11, 22]	<0.0001
mRs at 3 months	1 [0, 2]	5 [3, 6]	<0.0001
Glutamate at admission, μM	134.2 ± 112.5	227.1 ± 123.8	0.036
sTWEAK at admission, pg/mL	3143.2 ± 2677.1	5828.6 ± 3248.9	0.002
IL-6, pg/mL	20.9 ± 17.8	30.9 ± 18.3	0.743

TOAST, Trial of Org 10,172 in Acute Stroke Treatment; NIHSS, National Institute of Health Stroke Scale; GOT, glutamic-oxaloacetic transaminase; mRs, Mod. Rankin scale; sTWEAK, soluble tumor necrosis factor-like weak inducer of apoptosis; L-6, interleukin-6.

**Table 2:** Logistic regression model for demographic and clinical features. Dependent variable: poor outcome at 3 months.

**sTWEAK ≤ 2900 pg/mL**						
	**Not Adjusted**			**Adjusted**		
	**OR**	**CI 95%**	***p***	**OR**	**CI 95%**	***p***
Latency time	1.00	0.99–1.01	0.973	1.00	1.00–1.05	0.061
Age	1.06	1.03–1.08	<0.0001	1.01	0.97–1.06	0.601
Women	1.86	1.08–3.19	0.025	1.71	0.26–2.91	0.494
Smoking	0.36	0.16–0.84	0.018	0.86	0.38–9.02	0.443
Alcoholism	1.12	0.46–2.71	0.803	1.15	0.21–6.39	0.870
Atrial fibrillation	4.36	2.32–8.18	<0.0001	1.12	0.34–3.61	0.854
Temperature at admission	1.03	1.00–1.44	0.047	1.24	0.16–5.61	0.433
Glucose at admission	1.00	0.99–1.01	0.178	1.00	0.99–1.05	0.146
Leukocytes at admission	1.12	1.02–1.22	0.013	1.21	1.00–1.47	0.042
Cardioembolic	5.22	3.05–6.18	<0.0001	1.37	1.11–4.16	0.035
Lacunar	0.64	0.34–0.93	<0.0001	0.85	0.47–1.03	0.126
GOT at admission	0.98	0.96–1.01	0.170	0.98	0.94–1.03	0.514
NIHSS at admission	1.18	1.13–1.24	<0.0001	1.31	1.12–1.44	<0.0001
**sTWEAK > 2900 pg/mL**						
	**Not Adjusted**			**Adjusted**		
	**OR**	**CI 95%**	***p***	**OR**	**CI 95%**	***p***
Latency time	1.00	0.99–1.00	0.688	1.01	0.99–1.01	0.387
Age	1.04	1.02–1.06	<0.0001	0.95	0.87–1.04	0.292
Women	1.60	1.39–2.92	0.019	2.35	0.32–17.35	0.401
Smoking	0.89	0.48–1.65	0.703	0.83	0.03–21.34	0.908
Alcoholism	1.34	1.15–1.76	0.009	3.39	0.17–68.08	0.425
Atrial fibrillation	2.88	1.59–5.21	<0.0001	1.51	0.04–6.29	0.600
Temperature at admission	3.18	2.31–4.38	<0.0001	1.72	0.11–4.72	0.735
Glucose at admission	1.00	1.00–1.01	0.010	0.98	0.97–1.00	0.093
Leukocytes at admission	1.14	1.06–1.22	<0.0001	1.46	0.99–2.14	0.055
Cardioembolic	3.18	2.17–5.39	<0.0001	2.57	1.19–5.63	0.048
Lacunar	0.81	0.63–0.99	<0.0001	0.89	0.70–1.09	0.084
GOT at admission	0.64	0.56–0.71	<0.0001	0.41	0.28– 0.68	<0.0001
NIHSS at admission	1.27	1.21–1.33	<0.0001	1.14	1.00–1.31	0.006

sTWEAK, soluble tumor necrosis factor-like weak inducer of apoptosis; NIHSS, National Institute of Health Stroke Scale; GOT, glutamic-oxaloacetic transaminase.

**Table 3:** Logistic regression model for demographic and clinical features. Dependent variable: poor outcome at 3 months.

**No Leukoaraiosis**						
	**Not Adjusted**			**Adjusted**		
	**OR**	**CI 95%**	***p***	**OR**	**CI 95%**	***p***
Latency time	1.00	0.99–1.00	0.899	1.00	1.00–1.00	0.061
Age	1.04	1.02–1.06	<0.0001	1.02	0.98–1.04	0.295
Women	1.66	1.43–1.99	0.045	1.62	0.32–2.20	0.154
Smoking	0.46	0.27–0.79	0.005	0.55	0.22–1.35	0.189
Alcoholism	1.68	0.87–3.23	0.123	1.60	0.58–4.31	0.362
Atrial fibrillation	1.69	1.23–2.69	<0.0001	1.55	0.69–3.50	0.290
Temperature at admission	1.06	0.81–1.40	0.654	1.68	0.43–3.07	0.097
Glucose at admission	1.00	1.00–1.01	0.023	1.00	0.99–1.00	0.316
Leukocytes at admission	1.10	1.02–1.18	0.006	1.05	0.95–1.16	0.335
Cardioembolic	4.72	2.71–7.23	<0.0001	3.77	1.62–5.23	0.019
Lacunar	0.71	0.50–0.97	<0.0001	0.77	0.52–0.94	0.048
GOT at admission	0.95	0.93–0.97	<0.0001	0.95	0.92–1.02	0.105
NIHSS at admission	1.15	1.11–1.19	<0.0001	1.17	1.10–1.23	<0.0001
**Leukoaraiosis**						
	**Not Adjusted**			**Adjusted**		
	**OR**	**CI 95%**	***p***	**OR**	**CI 95%**	***p***
Latency time	1.00	0.99–1.00	0.407	0.99	0.99–1.00	0.556
Age	1.05	1.04–1.07	<0.0001	1.06	1.00–1.12	0.036
Women	1.46	1.31–1.67	<0.0001	1.23	0.36–4.22	0.075
Smoking	0.65	0.39–1.10	0.111	0.32	0.06–1.78	0.193
Alcoholism	1.89	1.07–3.34	0.028	1.16	0.21–6.35	0.864
Atrial fibrillation	3.97	2.35–6.71	<0.0001	4.42	0.85–6.37	0.270
Temperature at admission	2.84	2.17–3.75	<0.0001	2.49	0.92–6.72	0.071
Glucose at admission	1.00	1.00–1.01	<0.0001	1.00	0.99–1.00	0.711
Leukocytes at admission	1.18	1.11–1.26	<0.0001	1.26	1.01–1.56	0.039
Cardioembolic	3.29	1.58–6.03	<0.0001	3.58	1.23–6.19	0.038
Lacunar	0.58	0.39–0.91	<0.0001	0.69	0.43–0.90	0.024
GOT at admission	0.77	0.74–0.82	<0.0001	0.75	0.69–0.82	<0.0001
NIHSS at admission	1.27	1.21–1.32	<0.0001	1.14	1.03–1.25	0.008

sTWEAK, soluble tumor necrosis factor-like weak inducer of apoptosis; NIHSS, National Institute of Health Stroke Scale; GOT, glutamic-oxaloacetic transaminase.

## Data Availability

The statistical analysis plan is available upon request. The data bank is not available for legal and ethical reasons.

## Supplementary Materials

The following supporting information can be downloaded at: https://www.mdpi.com/article/10.3390/ijms25126554/s1.
