# Supplementary material for: Precision Medicine for Blood Glutamate Grabbing in Ischemic Stroke
Source: Int J Mol Sci. 2024 Jun 14;25(12):6554. doi: 10.3390/ijms25126554 (PMC11204254; doi:10.3390/ijms25126554)
Supplement: Supplementary file 1 [file ijms-25-06554-s001.zip › ijms-3033192-supplementary.pdf]

Supplementary material

Table S1. Bivariate analysis of clinical features according to exclusion criteria.

	**Excluded patients** n = 3,697	**Valid patients** n = 906	**p**
Latency time	240.9 ± 166.2	272.7 ± 193.1	0.314
Age, years	72.1 ± 13.9	72.7 ± 13.8	0.408
Female, %	45.1	48.7	0.106
Wake-up stroke, %	11.5	13.8	0.079
Pre-morbid mRS [IQR]	0 [0, 1]	0 [0, 1]	0.619
Temperature at admission, ºC	37.1 ± 0.6	37.2 ± 0.8	0.377
Glucose at admission, mg/dL	138.1 ± 58.3	145.9 ± 71.1	0.067
Leukocytes at admission, x10^3/mL	9.7 ± 3.2	9.8 ± 3.4	0.506
GOT at admission, U/L	29.2 ± 3.2	28.8 ± 3.4	0.214
Intravenous fibrinolysis, %	13.6	14.5	0.418
Thrombectomy, %	5.4	5.6	0.711
NIHSS at admission [IQR]	14 [8, 19]	13 [6, 20]	0.184
Mod. Rankin scale at 3 months [IQR]	3 [1, 4]	3 [2, 5]	0.077
TOAST			0.214
Atherothrombotic, %	22.0	20.5	
Cardioembolic, %	36.9	37.1	
Lacunar, %	9.0	9.4	
Indeterminate, %	30.8	31.1	
Other, %	1.3	1.9	
Leukoaraiosis			0.529
No, %	41.3	41.3	
Grade I, %	26.6	28.1	
Grade II, %	16.2	17.0	
Grade III, %	15.9	13.6	

NIHSS, National Institute of Health Stroke Scale: GOT, glutamic-oxaloacetic transaminase
sTWEAK, soluble tumor necrosis factor-like weak inducer of apoptosis; mRs, Mod. Rankin scale

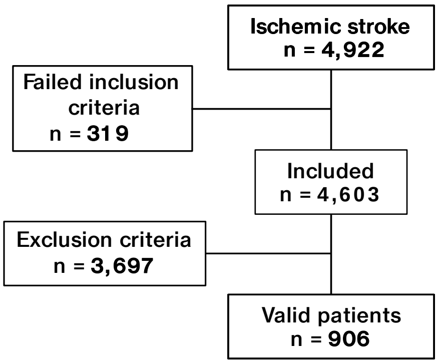

Figure S1 Flowchart of patient screening from BICHUS

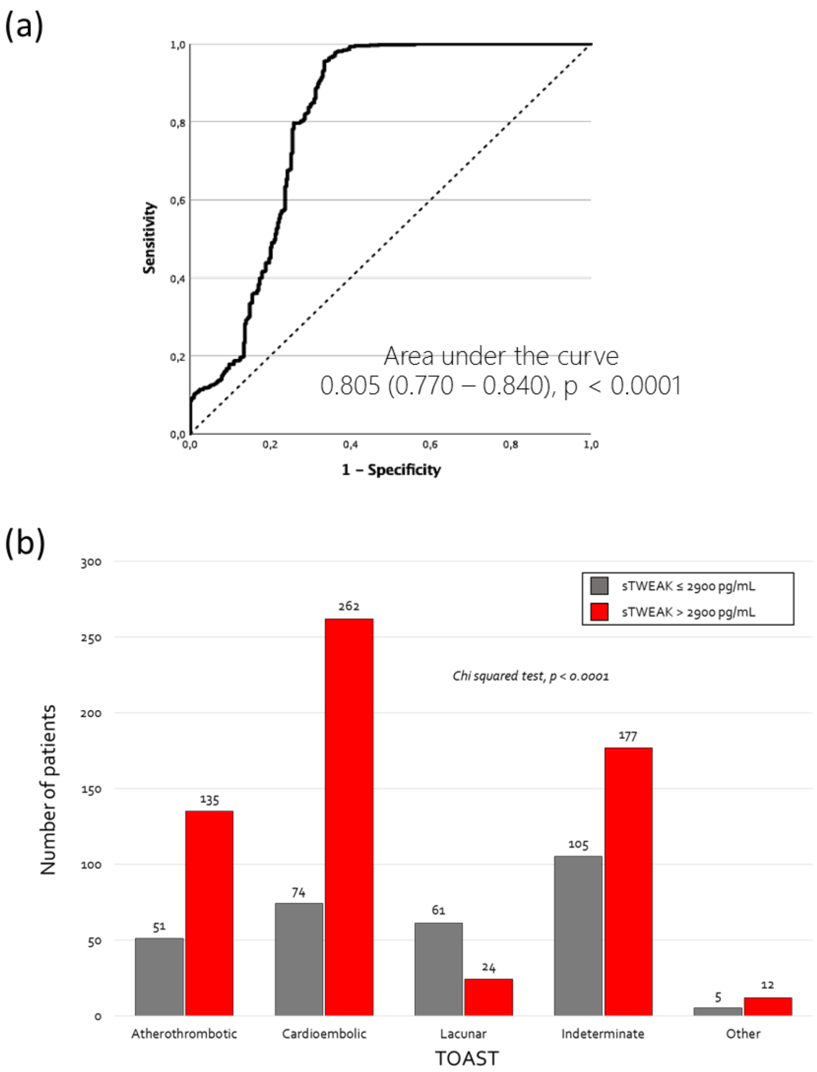

Figure S2 (a) Sensitivity vs specificity of soluble tumor necrosis factor-like weak inducer of apoptosis (sTWEAK) as biomarkers of poor outcome at 3 months. sTWEAK showed a sensitivity of 88%, specificity of 70%, and AUC of 0.805 (CI 95% 0.770 – 0.840), p < 0.0001). The cut-off for it was > 2,900 pg/mL. (b) Number of patients who suffer each etiology of ischemic stroke by sTWEAK serum level.

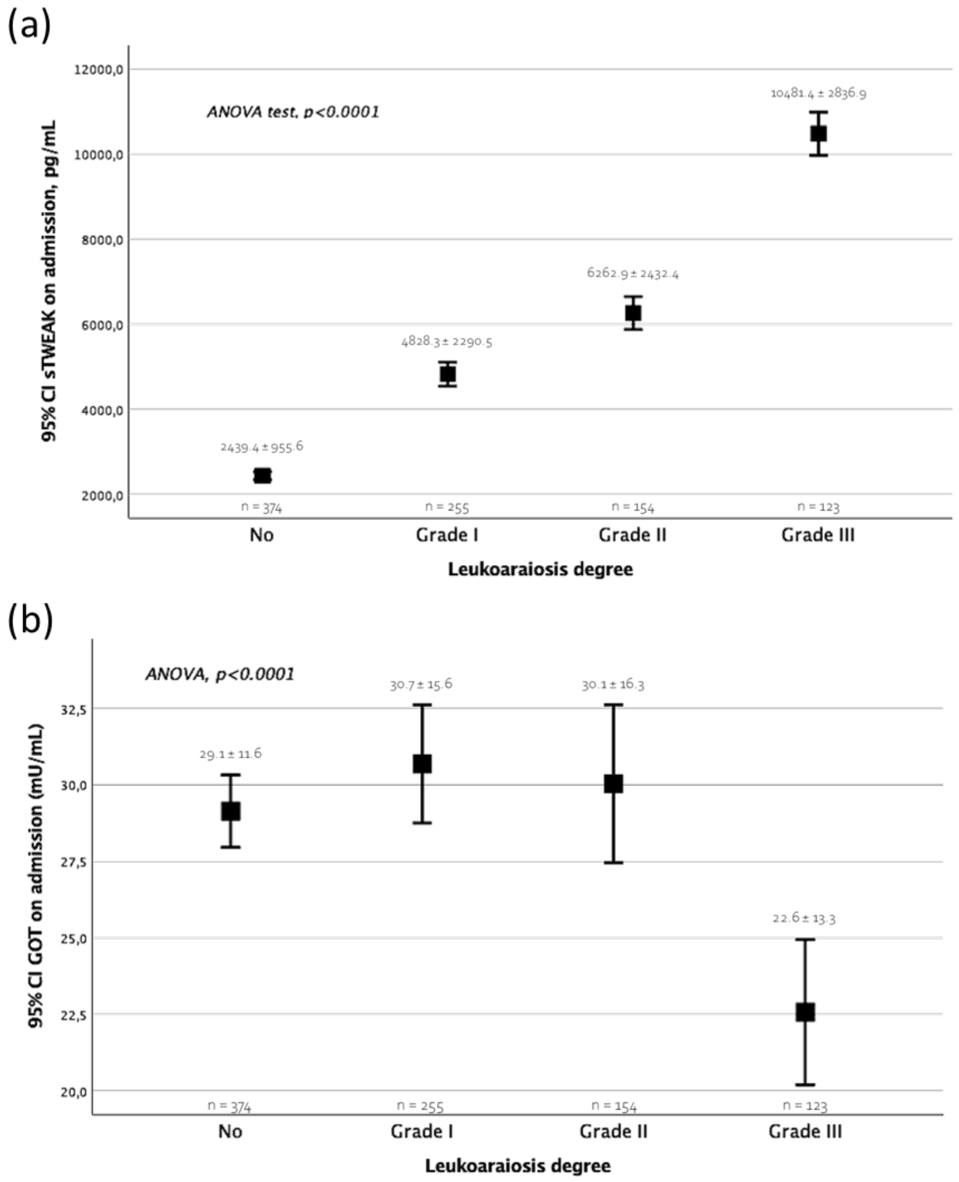

Figure S3 Relationship between the leukoaraiosis degree and (a) level of soluble tumor necrosis factor-like weak inducer of apoptosis (sTWEAK) and (b) glutamic-oxaloacetic transaminase (GOT) at admission.
